# High Blood Concentration of Leukocyte-Derived Extracellular Vesicles Is Predictive of Favorable Clinical Outcomes in Patients with Pancreatic Cancer: Results from a Multicenter Prospective Study

**DOI:** 10.3390/cancers14194748

**Published:** 2022-09-29

**Authors:** Davide Brocco, Domenico De Bellis, Pietro Di Marino, Pasquale Simeone, Antonino Grassadonia, Michele De Tursi, Tommaso Grottola, Fabio Francesco Di Mola, Patrizia Di Gregorio, Barbara Zappacosta, Antonio Angelone, Laura De Lellis, Serena Veschi, Rosalba Florio, Simone De Fabritiis, Fabio Verginelli, Marco Marchisio, Marta Caporale, Dimitri Luisi, Pierluigi Di Sebastiano, Nicola Tinari, Alessandro Cama, Paola Lanuti

**Affiliations:** 1 Department of Pharmacy, University “G. D’Annunzio” Chieti-Pescara, 66100 Chieti, Italy; 2 Department of Medicine and Aging Sciences, University “G. D’Annunzio” Chieti-Pescara, 66100 Chieti, Italy; 3 Center for Advanced Studies and Technology (C.A.S.T.), University “G. D’Annunzio” Chieti-Pescara, 66100 Chieti, Italy; 4 Clinical Oncology Unit, S.S. Annunziata Hospital, 66100 Chieti, Italy; 5 Department of Innovative Technologies in Medicine and Dentistry, University “G. D’Annunzio” Chieti-Pescara, 66100 Chieti, Italy; 6 Surgical Oncology Unit, Casa di Cura Pierangeli, 65124 Pescara, Italy; 7 Unit of Transfusion Medicine and Hematology, S.S. Annunziata Hospital, 66100 Chieti, Italy; 8 Anatomical Pathology Unit, Casa di Cura Pierangeli, 65124 Pescara, Italy; 9 Medical Oncology Unit, S. Spirito Hospital, 65124 Pescara, Italy; 10 Department of Medical, Oral & Biotechnological Sciences, University “G. D’Annunzio” Chieti-Pescara, 66100 Chieti, Italy

**Keywords:** pancreatic cancer, leukocytes, extracellular vesicles

## Abstract

**Simple Summary:**

Blood-circulating extracellular vesicles (EVs) are emerging as key players to develop novel liquid biopsy-based approaches in cancer, including pancreatic cancer. In this study, we aimed to explore the prognostic and predictive value of blood-circulating extracellular vesicles released by immune cells in patients with pancreatic cancer. A recently patented flow cytometry protocol was applied for the identification and phenotypical characterization of blood-circulating EVs in a cohort of 56 patients with pancreatic cancer (PC) and in a group of 48 healthy controls. We observed an increased blood concentration of leukocyte-derived EVs (CD45+) and PD-L1+ EVs in patients with PC as compared to healthy controls. Intriguingly, a high blood concentration of leukocyte-derived EVs identified PC patients with a good prognosis and improved clinical outcomes. This study revealed the promising role of EVs released by immune cells as a source of candidate biomarkers in patients with pancreatic cancer.

**Abstract:**

Pancreatic cancer (PC) is one of the leading causes of cancer-related death worldwide. Identification of novel tumor biomarkers is highly advocated in PC to optimize personalized treatment algorithms. Blood-circulating extracellular vesicles hold promise for liquid biopsy application in cancer. We used an optimized flow cytometry protocol to study leukocyte-derived EVs (CD45+) and PD-L1+ EVs in blood from 56 pancreatic cancer patients and 48 healthy controls (HCs). Our results show that PC patients presented higher blood levels of total EVs (*p* = 0.0003), leukocyte-derived EVs (LEVs) (*p* = 0.001) and PD-L1+ EVs (*p* = 0.01), as compared with HCs. Interestingly, a blood concentration of LEVs at baseline was independently associated with improved overall survival in patients with borderline resectable or primary unresectable PC (HR = 0.17; 95% CI 0.04–0.79; *p* = 0.02). Additionally, increased blood-based LEVs were independently correlated with prolonged progression-free survival (HR = 0.10; 95% CI 0.01–0.82; *p* = 0.03) and significantly associated with higher disease control rate (*p* = 0.02) in patients with advanced PC receiving standard chemotherapy. Notably, a strong correlation between a decrease in blood LEVs concentration during chemotherapy and disease control was observed (*p* = 0.005). These intriguing findings point to the potential of LEVs as novel blood-based EV biomarkers for improved personalized medicine in patients affected by PC.

## 1. Introduction

Pancreatic cancer (PC) is a major cause of cancer-related mortality worldwide, and its incidence and global burden are predicted to grow in the future decades [1]. Survival rates for pancreatic cancer are among the lowest for solid tumors, despite recent improvements leading to a 5-year survival rate lower than 10% in Europe and US [2]. Lack of appropriate screening and diagnostic tools, difficulties in biopsy and surgical procedures, aggressive tumor biology, as well as primary radiotherapy and chemotherapy resistance represent some of the key factors determining such dismal prognosis in patients with PC [3]. Increasing understanding of PC biology and identification of biological markers capable to predict tumor behavior, as well as treatment response, are critically important to foster advancements in PC clinical management, which may result in survival improvements for patients [4,5]. In this regard, considering difficulties in tumor tissue sampling due to the anatomic location of the pancreas, the application of non-invasive circulating tumor biomarkers for PC early diagnosis, recurrence prediction, and treatment selection is strongly advocated [6]. Several molecules have been explored as biochemical markers in PC but serum carbohydrate 19-9 antigen (CA 19-9) is the only blood-based biomarker approved by the FDA to support PC diagnosis and monitoring [7]. Nevertheless, limited specificity and sensitivity due to non-specific expression in several benign and malignant diseases, as well as the risk of false negative tests in individuals with Lewis negative genotype, hamper the universal applicability of serum CA 19.9 in PC patients [8]. Taken together, there is an urgent unmet need for novel biomarkers that might be helpful to optimize decision algorithms and improve treatment strategies for this deadly disease.

Extracellular vesicles (EVs) are bilayer membrane-bound particles and are released by almost all cell types to mediate intercellular communication. EVs are crucial for several pathophysiological processes associated with PC initiation, progression, and metastasis [9]. Furthermore, EVs have been involved in mechanisms related to tumor microenvironment (TME) remodeling, as well as in the crosstalk among cancer cells, immune cells, and other non-immune host cells participating in the TME complexity [10]. The dynamic tumor-immune system interplay can be partly mediated by EVs, which have the potential to enhance the immune response against malignant cells or to favor immunosuppressive TME modifications [11]. In PC, tumor immune response can be regulated by EVs released both by cancer cells and other TME-associated cell types, including leukocytes [12]. In addition, EVs released by leukocytes within pancreatic tumors were shown to regulate chemoresistance, tumor proliferation, invasion, and metastasis [13,14,15]. Since EVs secreted within the tumor can reach the bloodstream, circulating tumor-induced EVs hold great promise as dynamic sources of biomarkers to be used as a platform for liquid biopsy-based approaches in cancer, including PC [16]. In this respect, although several studies have focused their attention on circulating pancreatic cancer cell-derived EVs [17,18,19], the analysis of blood-based EVs secreted by immune cells, and the correlation between leukocyte-derived EVs (LEVs) and patient clinical outcomes is lacking in pancreatic cancer. Furthermore, circulating EVs expressing immune checkpoint molecules (ICMs) have been associated with tumor immune regulation, treatment response, and survival in cancer patients [20,21,22]. However, the biological and clinical value of blood-circulating EVs expressing ICMs has been poorly explored in PC [12,23].

Recently, we developed and patented a polychromatic flow cytometry (FC) method for EV phenotyping as a simple, rapid, and solid protocol to study different EV subtypes in fresh peripheral blood samples [24,25,26,27,28]. In the present study, we combined this FC-based method for EV identification with the evaluation of EV surface expression of the pan-leukocyte antigen CD45 to identify and enumerate blood-circulating leukocyte-derived EVs in a cohort of patients with PC at different stages of the disease. In addition, we evaluated the expression of the ICM programmed death ligand 1 (PD-L1) both on blood-circulating immune- and non-immune-derived EVs. We compared EV concentrations between PC patients and healthy controls and evaluated the correlation between EV blood levels with patient clinical characteristics. Then, we investigated the prognostic and predictive value of total EVs, LEVs, and PD-L1+ EVs in a cohort of patients with borderline resectable or primary unresectable PC.

## 2. Materials and Methods

### 2.1. Patients

Adult patients with a histologically or cytologically confirmed diagnosis of malignant epithelial tumors of the pancreas were enrolled in this prospective observational study. Patients were recruited from the Clinical Oncology Units of the “SS Annunziata” Hospital in Chieti (Italy), the “S. Spirito” Hospital in Pescara (Italy), and the Surgical Oncology Unit of the medical center “Casa di Cura Pierangeli” in Pescara (Italy). Patients were recruited from September 2020. A cohort of age and sex-matched healthy controls was also included in the study. Demographic and clinicopathological variables including Eastern Cooperative Oncology Group (ECOG) performance status (PS), age, sex, weight, height, primary tumor location, tumor grading, clinical stage, serum CA 19.9, neutrophil to lymphocyte ratio (NLR), number and location of metastatic sites, blood total leukocyte, and neutrophil and lymphocyte counts were collected at baseline. Clinical stage was evaluated according to the eighth edition of AJCC/UICCA. All procedures involving human participants were carried out in accordance with the ethical standards of the 1964 Helsinki declaration and its later amendments or with a comparable ethical standard. This study was approved by the local ethics committee on 25 February 2016. All patients gave written informed consent.

### 2.2. Blood Collection

A baseline peripheral blood sample was drawn at study enrollment. A second blood sample was collected after 8 (+/−4) weeks from day 1 of chemotherapy in patients who were candidates for systemic therapy for advanced disease. Peripheral blood was harvested (21 G needles) in two sodium citrate tubes. The first tube collected was discarded to avoid analysis of EVs released as a result of vascular damage. Blood samples were processed within 4 h from venipuncture.

### 2.3. Flow Cytometry Detection of Extracellular Vesicles

Lipophilic cationic dye (LCD), phalloidin, and reagents listed in Supplementary Table S1 were mixed in a PBS solution. Then, 5 μL of whole blood was added to the mix. Centrifugation (21,000× *g*, 10 min, 4 °C) of each reagent stock solution was performed before the use to minimize immune complex formation and to reduce unspecific background triggered by aggregation of reagents. Each sample was incubated at RT in the dark for 45 min and then diluted with 500 μL of PBS 1×. After sample dilution, 1 × 10^6^ events/sample were immediately acquired with BD FACSVerse flow cytometer (BD Biosciences, San Jose, CA, USA). Volumetric count was applied to calculate EV concentrations. The trigger threshold was set on the fluorescent channel (APC) in which LCD emits, as previously reported [27,29]. The signal pulse height (H) was employed for scatters and any fluorescent signals. Megamix-Plus beads (Byocitex, Marseille, France) and the Rosetta Calibration System (Exometry, Amsterdam, The Netherlands) were used to define and verify EV scatter gates over time, as previously reported [27,29]. Gating strategy and non-specific fluorescence were defined by fluorescence minus one (FMO) controls [30]. Reagent-only, buffer-only, and 1% Triton X-100 controls were performed for improved discrimination of EV population from contamination and debris. Data were analyzed using FlowJo v.10.8 (BD Biosciences, San Jose, CA, USA).

### 2.4. Flow Cytometry Subtyping of Extracellular Vesicles

EVs were identified as LCD+/phalloidin− events, which fell in the scatter area with physical parameters lower than platelets (Supplementary Figure S1A,B). Size and morphological characterization of EVs identified by the LCD-based protocol was detailed in previous reports [27,29]. Marchisio et al,. previously reported that up to 90% of blood-based LCD+/phalloidin-particles presented a diameter larger than 160 nm [27], which according to MISEV 2018 guidelines [31], are referred as “extracellular vesicles” (EVs). Total EVs (LCD+/phalloidin− events) were analyzed on a CD45-H/CD133-H dot plot and CD45+ events were gated (Supplementary Figure S1C). The total EV population was then analyzed on a CD45-H/PD-L1-H dot plot, and PD-L1+CD45+ event, as well as PD-L1+CD45- event, EVs were identified (Supplementary Figure S1D).

### 2.5. Statistical Analysis

Statistical analysis was performed using SPSS v23.0 (IBM SPSS, Chicago, IL, USA) and Stata v.17 (StataCorp, College Station, TX, USA). No assumption of normality of the data was formulated. Thus, non-parametric tests were used for comparisons. Continuous variables were compared by applying the Mann–Whitney *U* test. The neutrophil-to-lymphocyte ratio (NLR) was calculated by dividing neutrophil counts by lymphocyte counts. BMI was calculated by dividing weight in kilograms by height in meters squared. Correlations between clinical–pathological variables and EV concentrations were analyzed by Spearman’s rank correlation coefficients. Univariate and multivariate Cox proportional hazards models were applied to calculate the hazard ratio (HR) together with 95% confidence intervals (CIs) for overall survival (OS) and progression-free survival (PFS). Univariate and multivariate exact logistic regression models were used to determine Odd Ratios and 95% confidence intervals (CIs) for disease control. Cut-offs for EV concentrations were calculated according to survival outcome by employing the Charité Cutoff Finder functions [32]. Median overall survival (mOS) was calculated using the Kaplan–Meier (KM) curve estimator. Differences in mOS were assessed using the log-rank test. Tumor response was evaluated according to RECIST 1.1. Disease control rate (DCR), defined as the percentage of patients who achieved stable disease, partial response (PR), or complete response (CR) was analyzed. DCRs were compared using Fisher’s exact test. The relative variation in EV concentration between first and second blood samples was calculated as fold change (EV concentration week 8/EV concentration week 0). Increasing EV concentration was defined as >25% increase in EV concentration from baseline condition (log_2_ fold change >0.32). Stable or decreasing EV concentration was set as any ≤25% increase or any decrease in EV concentration from baseline condition (log_2_ fold change ≤0.32). The SPSS biased-corrected and accelerated bootstrap method with 1000 bootstrap samples and a 95% confidence interval was employed for internal validation. A *p*-value of <0.05 was considered statistically significant.

## 3. Results

### 3.1. Clinical Characteristics of PC Patients Enrolled

From September 2020 to May 2022, 56 patients with PC were enrolled in the study. Overall baseline demographic and clinical features of patients included in the study are summarized in Table 1 and Table 2. Thirty-one patients (55.4%) were metastatic at the time of enrollment, whereas 25 of 56 (44.6%) patients were diagnosed with localized PC (stage I–III). Patients with primary unresectable PC for locally advanced or metastatic disease (*n* = 32) and those with borderline resectable PC (*n* = 15) received chemotherapy-based systemic therapy after being included in the study. Nine patients (16.1%) were primarily treated with surgery. The median follow-up time was 5.1 (95% CI 3.8–7.2) months in the cohort of patients with primary unresectable or borderline resectable PC, whereas the overall one-year OS was 54%.

### 3.2. Patients with PC Present Increased Blood Levels of Leukocyte-Derived and PD-L1+ EVs

**Figure 1:**
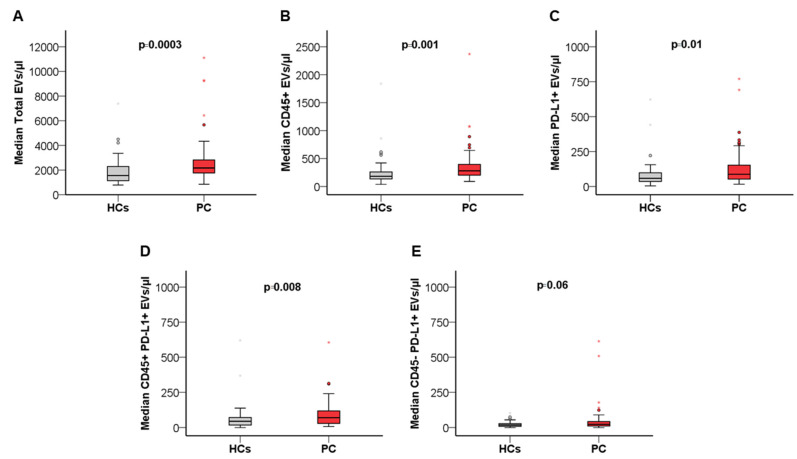
Box plots showing differences in total circulating EVs (**A**), CD45+ EV (**B**), PD-L1+ EV (**C**), CD45+PD-L1+ EV (**D**), and CD45-PD_L1+ EV (**E**) concentration between patients with PC (*n* = 56) and healthy controls (*n* = 48). Statistical comparison was performed by applying the Mann–Whitney *U* test. Circles and stars represent outliers. For visual clarity, highest extreme values are not shown.

Median blood concentrations of total EVs, leukocyte-derived (CD45+) EVs, and PD-L1+ EVs were calculated and compared between the overall cohort of patients with PC (*n* = 56) and a group of age and sex-matched HCs (*n* = 48) (Table 3; Figure 1).

We combined PD-L1 and CD45 staining to identify and enumerate blood-circulating PD-L1+ EVs of leukocyte (CD45+PD-L1+) and non-leukocyte (CD45-PD-L1+) cellular origin. Blood concentrations of different subsets of PD-L1 expressing EVs were compared between patients with PC and HCs. Notably, blood levels of total EVs and leukocyte-derived EVs were significantly higher in PC patients as compared to HCs (1.4-fold increase, *p* = 0.0003; 1.5-fold increase, *p* = 0.001, respectively) (Table 3; Figure 1). Similarly, a 1.5-fold increase in blood concentrations of PD-L1+ and CD45+PD-L1+ EVs was observed in the PC cohort (*p* = 0.01; *p* = 0.008, respectively). Conversely, no significant difference in median blood concentration of CD45-PD-L1+ EVs was found (Table 2; Figure 1). In the cohort of patients with PC primarily treated with surgery (*n* = 9), blood concentrations of total EVs (median EVs/µL = 2483.7; CI 95% 1885.6–2984.4), as well as CD45+ (median EVs/µL = 233.3; CI 95% 218.4–357.1), PD-L1+ (median EVs/µL = 70.0; CI 95% 43.7–303.3), CD45+PD-L1+ (median EVs/µL = 57.6; CI 95% 21.3–126.0) and CD45-PD-L1+ EVs (median EVs/µL = 28.6; CI 95% 12.4–177.3) were in line with blood levels of EV populations observed in the overall PC cohort.

### 3.3. Blood-Circulating PD-L1+ and PD-L1+CD45+ EVs Are Associated with Site of Metastasis in Patients with PC

Correlations between blood-derived EVs and clinical–pathological factors including ECOG PS, age, sex, BMI, primary tumor location, tumor grading, clinical stage, serum CA 19.9, neutrophil to lymphocyte ratio (NLR), and number and location of metastatic sites were assessed in the overall patient cohort (Supplementary Table S2). Notably, blood concentrations of PD-L1+ and CD45+PD-L1+ EVs were significantly and negatively correlated with peritoneal carcinomatosis (Supplementary Table S2). Differently, a positive correlation was found between liver metastasis and blood concentrations of PD-L1+ and CD45+PD-L1+ EVs. No further correlations were found between the examined clinical–pathological factor and other EV populations. Considering the relationship observed between circulating PD-L1+ EVs and the site of metastasis, we compared median blood concentrations of CD45+PD-L1+ and CD45-PD-L1+ EV in stage IV PC patients stratified according to liver and/or peritoneal metastatic dissemination. Of note, patients with liver metastasis presented higher levels of CD45+PD-L1+ EVs, as compared with stage IV patients without liver metastasis (median CD45+PD-L1+ EVs/µL (95% CI) = 89.9 (54.9–145.3) vs. 28.6 (23.3–91.5); *p* = 0.02), whereas a significant decrease in the median concentration of this EV subset was detected in patients with peritoneal carcinomatosis, as compared to those without peritoneal metastatic dissemination (median CD45+PD-L1+ EVs/µL (95% CI) = 27.8 (10.3–76.4) vs. 96.0 (54.9–119.1); *p* = 0.007) (Supplementary Figure S2). Blood levels of CD45-PD-L1+ EVs were not statistically different between patients with or without liver metastasis, as well as with or without peritoneal metastasis.

### 3.4. High Blood Levels of CD45+ EVs Independently Predict Improved Survival in Patients with Borderline Resectable and Primary Unresectable PC

We investigated whether blood-circulating EV concentrations at treatment baseline were associated with survival in patients with metastatic, locally advanced, and borderline resectable PC (*n* = 47). Univariate and multivariate Cox proportional hazards regression analyses were used to explore the correlation between patient survival and pre-treatment blood levels of total EVs, as well as EV subpopulations. Cut-off values for survival analysis were calculated for the whole EV population and EV subtypes, as described above (Supplementary Figure S3). On univariate analysis, blood concentrations of total EVs (*p* = 0.04), CD45+ (*p* = 0.01), PD-L1+ (*p* = 0.02), CD45+PD-L1+(*p* = 0.02), and CD45+PD-L1+(*p* = 0.04) EVs were significantly associated with patient survival (Table 4). Univariate Cox proportional hazards regression analysis was used to evaluate the association between OS and clinical–pathological factors including ECOG PS, age, number and site of metastasis, BMI, tumor grading, primary tumor location, NLR, serum CA 19.9, and clinical stage (Table 4). In this regard, higher NLR and peritoneal metastasis significantly correlated with a higher risk of death in the study cohort (*p* = 0.01; *p* = 0.02, respectively). Cox regression univariate analyses were confirmed via bootstrap validation. All variables significantly correlated with OS (*p* < 0.05) in the univariate analysis including total EVs, CD45+, PD-L1+, CD45+PD-L1+, CD45-PD-L1+ EVs, NLR, and peritoneal metastasis were selected as candidate variables for the multivariate analysis. A Cox proportional hazards regression multivariate analysis using a stepwise backward procedure was used to derive a final model of the variables that had a significant independent relationship with survival. In this model, a variable is stepwise removed if the corresponding *p* value is >0.10. Intriguingly, in the final multivariate stepwise model, only NLR and blood levels of CD45+ EVs were independently associated with survival (Table 4).

**Figure 2:**
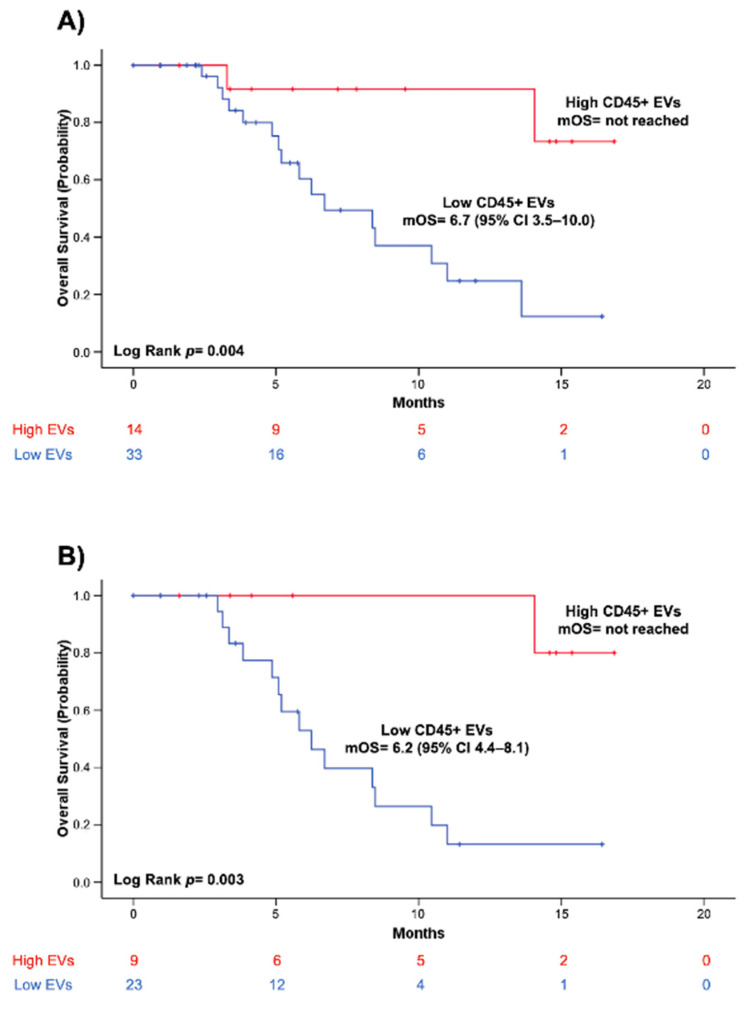
Kaplan–Meier (KM) curves showing the relationship between overall survival and blood concentration at baseline of leukocyte-derived EVs (CD45+) in the cohort of patients with borderline resectable, locally advanced, or metastatic PC (**A**) and in the subgroup of patients with locally advanced or metastatic PC (**B**). Log-rank test was used to statistically compare KM survival curves.

The relationship between overall survival and blood concentrations of CD45+ EVs is depicted in Figure 2. Kaplan-Meier (KM) survival curves showed that patients with higher baseline levels of CD45+EVs (>379.1 EVs/μL) presented remarkably prolonged survival, as compared to patients with lower CD45+ EV concentration (*p* = 0.004) (Figure 2A). Of note, a significant correlation between longer survival and higher CD45+ EVs concentration was also observed in the sub-group of patients with metastatic or locally advanced PC (*n* = 32) (*p* = 0.003) (Figure 2B).

### 3.5. Increased Blood Levels of Circulating CD45+ EVs Are Associated with Higher Disease Control Rate and Longer Progression-Free Survival in Patients with Advanced PC

To investigate whether blood EV concentrations at baseline correlated with treatment response, we analyzed progression-free survival and disease control rate in patients with locally advanced or metastatic PC (*n* = 32). Data for PFS analysis were available in 30 of 32 patients. Median PFS was 5.7 months (95% CI 4.0–7.5). Radiological assessment of treatment response was available in 20 of 32 patients. The overall DCR was 65%.

**Figure 3:**
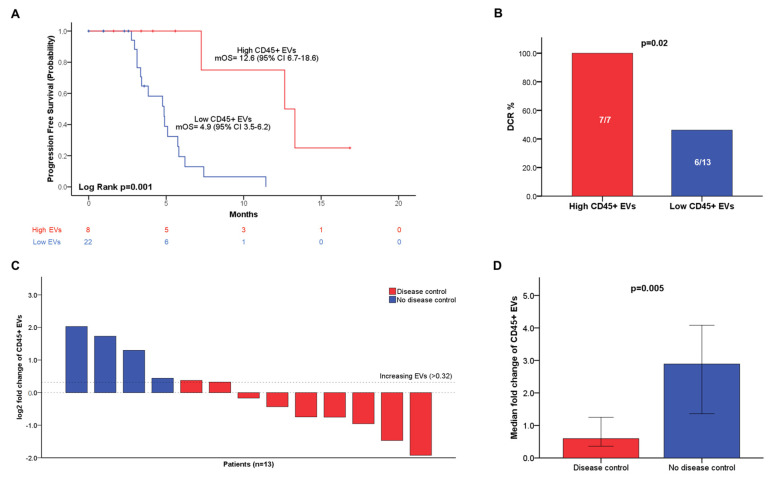
Kaplan–Meier (KM) curves illustrating the association between progression-free survival and blood concentration at baseline of leukocyte-derived EVs (CD45+) in the cohort of patients with locally advanced or metastatic PC (**A**); Histograms showing disease control rate in patients with high (red column) and low (blue column) CD45+ EVs (**B**); Waterfall plot depicting distributions of patients with disease control (red column) or progressive disease (blue column) after standard chemotherapy according to variations in blood concentrations of leukocyte-derived EVs (CD45+) during treatment (**C**); Bar charts with error bars showing difference in median fold change in leukocyte-derived EVs (CD45+) in patients with disease control (red bar) or progressive disease (blue bar) (**D**). Error bars represent 95% confidence intervals. Log-rank test was used to statistically compare KM survival curves. DCRs were compared using Fisher’s exact test. Fold changes were compared using Mann–Whitney *U* test.

Univariate and multivariable Cox proportional hazards regression analyses were used to explore the correlation between PFS and pre-treatment blood levels of total EVs and EV subpopulations. EV concentrations were dichotomized using the same cut-off values employed for OS analysis. On univariate analysis, blood concentrations of total EVs (*p* = 0.03), CD45+ (*p* = 0.01), PD-L1+ (*p* = 0.02), and PD-L1+CD45-(*p* = 0.02) EVs were significantly associated with PFS (Supplementary Table S3). Univariate analysis was also performed to study the association between PFS and patients’ characteristics, including ECOG PS, number and site of metastasis, tumor grading, NLR, CA 19.9, line of systemic therapy, and type of chemotherapy regimen (Supplementary Table S3). On univariate analysis, significant correlations between PFS and NLR (*p* = 0.006), peritoneal (*p* = 0.02), and liver metastasis (*p* = 0.03) were found. Of note, no difference in PFS was observed according to the employed chemotherapy regimen and line of systemic therapy. Multivariate Cox regression analysis was then performed including variables that significantly correlated with PFS (*p* < 0.05) on univariate analysis. On multivariate analysis, the blood concentrations of CD45+ EVs (*p* = 0.03) and NLR (*p* = 0.03) were independently associated with PFS. In Figure 3A, KM curves showed the association between increased blood levels of circulating CD45+ EVs (>329 EVs/μL) and improved mPFS (*p* = 0.001).

Given the small sample size of the cohort (*n* = 20), exact logistic regression was employed to evaluate the correlation between EV blood concentrations at baseline and disease control, which was defined as the occurrence of stable disease, partial or complete response after systemic therapy (Supplementary Table S4). Intriguingly, blood-circulating CD45+ EVs were significantly associated with disease control (OR = 0.11 (95% CI 0.00–0.95); *p* = 0.04), whereas no significant correlation was found for total EVs, nor other EV subtypes (Supplementary Table S4). We then evaluated differences in disease control according to the stratification of patients in groups with high or low CD45+ EV concentrations using the cut-off value of 379.1 EVs/μL. Strikingly, all patients (DCR = 100%) with high CD45+ EVs achieved disease control, whereas disease progression occurred in more than half of PC patients with low concentrations of blood derived CD45+ (DCR = 46.2%) (*p* = 0.02) (Figure 3B).

### 3.6. Blood-Circulating CD45+ EV Dynamics Predicts Disease Control in Patients with Advanced PC

To examine whether variations in blood levels of the selected EV populations could play a role as predictors of treatment resistance, we correlated the occurrence of disease control after systemic chemotherapy with modifications in blood-circulating EV concentrations between baseline blood samples collected on day 1 (+/− 2 weeks) of chemotherapy, and on-treatment blood samples drawn after 8 weeks (+/− 4 weeks). On-treatment blood samples were collected in 13 of 20 advanced PC patients with an available radiological assessment of treatment response. Fold changes between baseline and on-treatment blood concentrations of total EVs, CD45+, PD-L1+, CD45+PD-L1+, as well as CD45-PD-L1+ EVs, were calculated and correlated with disease control by univariate exact logistic regression analysis (Supplementary Table S5). Intriguingly, a significant positive correlation between the increasing fold change in CD45+ EVs and disease progression was found (*p* = 0.003). Of note, the median fold change in CD45+EVs was significantly reduced (fold change = 0.59 95% CI 0.36–1.07) in patients achieving disease control, as compared with a 2.88 (95% CI 1.36–4.08) fold change observed in patients with progressive PC (*p* = 0.005) (Figure 3C,D). Disease control occurred in the whole population of patients with stable or decreasing CD45+EV concentrations (8/8), whereas most patients with increasing CD45+ EVs (4/5) experienced disease progression (*p* = 0.007) (Figure 3C) (Supplementary Figure S4). No correlation was found between disease control and changes in blood-circulating total EVs, nor in PD-L1+ EVs (Supplementary Table S6).

## 4. Discussion

Liquid biopsy is emerging as a noninvasive and convenient approach for cancer diagnosis, prognosis, and monitoring. The detection of circulating tumor cells (CTCs), circulating tumor DNA (ctDNA), or cell-free tumor RNA in different body fluids (i.e., peripheral blood, saliva, or urine) represents a significant step forward with respect to surgical or puncture biopsy [33]. In the last decades, emerging evidence has indicated a pivotal role of EVs in cancer biology. Recent technological advancements in the EV field have improved the capability to detect, isolate, and characterize tumor-induced EVs circulating in different biofluids, thus promoting the application of EVs as a valuable source for liquid biopsy [34]. We have previously patented an optimized flow-cytometry-based method for simple and rapid flow cytometry EV phenotyping from fresh peripheral blood samples [24,25,26,27,28]. By combining the above-mentioned protocol for EV analysis and appropriate flow cytometry panels for the study of selected EV surface markers, it is possible to identify and enumerate blood-circulating EVs of different cellular origins [27,31,35]. Here, considering growing evidence for the role of EVs secreted by immune cells in PC, we applied this innovative FC-based method to profile leukocyte-derived EVs to evaluate EV expression of the immune checkpoint molecule PD-L1 in blood samples of patients affected by pancreatic cancer at different stages of the disease.

Interestingly, our data showed that the concentration of total circulating EVs was significantly higher in the group of PC patients as compared with the cohort of healthy controls. Of note, a similar increase in blood whole EV compartment as compared with healthy donors was previously reported in patients with other malignancies [24,25,26,36,37,38]. Expansion of the total EV population can be related to increased secretion of EVs promoted by the tumor [39]. Notably, this finding shows how tumors are capable of significantly perturbating the circulating EV burden; although, characterization of EV phenotype and cargo is necessary to understand how single EV subpopulations contribute to these systemic alterations in patients with PC. In this regard, our data showed that the whole population of CD45+ EVs was significantly increased in PC patients, as compared with healthy controls. In a previous study, Nanou et al. analyzed LEVs in blood samples of patients with metastatic prostate, breast, colorectal, and non-small cell lung cancer [40]. In that report, the authors did not observe any difference in blood levels of LEVs between cancer patients and healthy donors, but the EV identification and counting method was based on an optimized cell search protocol, which is different from the one used in the present work. It is conceivable that the increase in circulating LEVs we observed in our work may reflect methodological differences between the studies, or a peculiar characteristic of patients with PC, not associated with the tumors investigated by Nanou et al. Larger studies enrolling patients with different cancer types including pancreatic cancer and applying the same experimental settings for EV identification, as well as subtyping, are needed to further investigate this issue. Our findings suggest that the increased CD45+ population of circulating EVs in patients with PC may represent the result of enhanced EV secretion by immune cells promoted by the tumor. Notably, it has been reported that plasma EVs primarily derive from whole blood cells, such as platelets and leukocytes, and only a remarkably small percentage of circulating EVs present biological features indicating a non-hematopoietic tissue origin, including cancer [41,42,43]. In a recent report, Koliha et al. found that less than 1% of plasma EVs originated directly from tumor tissue in a cohort of patients with melanoma [43]. Furthermore, the authors observed altered signals for platelet and leukocyte markers in plasma EVs of melanoma patients, as compared with healthy controls. In the present study, we observed a greater than 50% increase in the whole LEV subpopulation in PC patients. Thus, it is likely that these changes in blood-circulating LEVs in patients with PC may be induced by altered EV secretion in circulating whole blood leukocytes and lymphoid structures within or outside the tumor, in parallel with enhanced EV production in tumor-infiltrating leukocytes [44,45]. However, further phenotypical and cargo characterizations of blood-based CD45+ EV populations are needed to elucidate which cell types or tissue mainly drive the expansion of the blood compartment of LEVs in PC.

Furthermore, we observed a significant increase in circulating PD-L1+ and PD-L1+ leukocyte-derived (CD45+PD-L1+) EVs in PC patients. However, no significant differences in terms of circulating concentrations of non-leukocyte PD-L1+ (CD45-PD-L1+)-derived EVs were found. Thus, the increase in PD-L1+ EVs in the peripheral blood of PC patients is mainly supported by the leukocyte compartment. Circulating EV-associated PD-L1 is involved in tumor immunosuppression and drug resistance [20,21]. The expression of PD-L1 on circulating EVs and its correlation with clinical outcomes were previously explored in patients with different cancer, including PC [21,22,23]. Here, we evaluated the combination of expression of a pan-leukocyte marker (CD45) and PD-L1 allowing an original window of observation. Specifically, we observed that the majority of blood-circulating PD-L1+ EV co-expressed CD45, suggesting an immune cellular origin for these vesicles. This finding should be considered when analyzing the whole PD-L1 EV population in blood samples. In addition, we found that the blood concentration of PD-L1+CD45+ EVs was higher in patients with liver metastases, whereas a lower level of this EV subset was significantly associated with peritoneal metastatic dissemination in patients with stage IV PC. Interestingly, analysis of tissue PD-L1 expression in liver vs. peritoneal metastasis in other gastrointestinal tumors appears in line with our findings in circulating EVs, suggesting that in our study metastases may contribute to the phenotypical characteristics of the blood EV compartment [46,47,48].

Furthermore, on univariate survival analysis, high blood concentrations at baseline of total EVs, leukocyte-derived EVs (CD45+), PD-L1+ EVs, PD-L1+CD45+, and PD-L1+CD45- were significantly associated with prolonged survival in a cohort of patients with borderline resectable or primary unresectable PC. More interestingly, multivariate analysis confirmed in our hand an independent prognostic role only for blood-circulating CD45+ EVs. Additionally, while assessing the predictive value of EV populations in the cohort of patients with advanced PC treated with systemic standard chemotherapy, we observed that a high blood concentration of LEVs at enrollment was independently associated with longer PFS and a strikingly high disease control rate. To our knowledge, this is the first report investigating the prognostic and predictive value of the whole blood compartment of LEVs evaluated as CD45+ events in a cohort of patients with pancreatic cancer. Notably, leukocyte-derived EVs are a heterogeneous population including both innate and adaptive immune cell-derived EVs, which are known to exhibit both immunostimulatory and immunosuppressive properties in cancer [49,50]. Immune cell-derived EVs released by activated T cells, dendritic cells, B cells, and NK cells are known to promote antitumoral immune responses and enhance tumor killing [50]. In this regard, the neutrophil-to-lymphocytes ratio, determined by the absolute counts of neutrophils and lymphocytes, may provide information about the balance between the systemic pro-tumoral inflammatory state and the anti-tumoral immune response [51]. However, in our study, we did not find any correlations between the blood concentration of LEVs and total leukocyte count, or NLR. Furthermore, both NLR and blood-circulating LEVs were here independently associated with OS. As reported by Auber M et al., it is worth noting that EV secretion rates (defined as the number of EVs secreted per minute by a cell) of whole blood cells are remarkably different across cell types. For this reason, whole blood cell-associated markers are not easily comparable with blood EV-based biomarkers, and it may not be possible to predict variations in the whole blood concentration of LEVs just by focusing on the characterization and enumeration of different blood cell types. In this regard, considering that CD45 is expressed on leukocytes with distinct roles in immunity, a deeper phenotypic lineage characterization to dissect the LEV subpopulation composition would be of interest for future investigation [41,52]. In particular, further phenotypic characterization of LEV subpopulations would be helpful to improve our understanding of the link between a high concentration of immune cell-derived EVs and improved clinical outcomes that we observed in patients with PC. Furthermore, in our hands, a high blood concentration of CD45+ EVs at baseline would predict disease control achievement with 100% sensitivity; although, this was in a small sample size cohort of patients with advanced PC. Considering the growing role of neoadjuvant treatment strategies in PC, the discovery of highly sensitive predictive biomarkers is emerging as crucial to identify those patients who could present a higher probability to respond to chemotherapy before surgery, thus optimizing treatment algorithms and improving clinical outcomes [53]. Thus, the present study indicates the potential predictive role of baseline blood concentrations of circulating LEVs in larger studies enrolling PC patients who are candidates for primary standard chemotherapy, including those with localized PC.

In this study, blood-circulating EVs were evaluated both at baseline and during standard chemotherapy. Interestingly, the median fold change in circulating leukocyte EV concentrations in patients with advanced PC achieving disease control during standard chemotherapy was significantly lower, as compared with patients with progressive PC. Remarkably, all patients who achieved disease control after systemic therapy presented stable or decreasing blood-circulating LEVs at a relatively early time point (8 weeks), highlighting a very high correspondence between treatment response and EV variations. Conversely, the majority of patients experiencing disease progression had increasing CD45+ EVs. Therefore, early variation in LEV blood concentration after therapy appears to reflect progression. At present, circulating CA19-9 levels are generally used to monitor treatment responses in PC, but, considering the limitations of this marker, more specific and sensitive non-invasive biomarkers are advocated to support the design of more personalized treatment strategies. Our results suggest that tracking blood concentration of CD45+ EV might be used to monitor therapy responses of pancreatic cancer patients, as blood levels of LEVs significantly decreased in patients who achieved disease control, but not in those with progressive disease.

## 5. Conclusions

Altogether, these results indicate that leukocyte-derived EVs might represent novel blood-based EV biomarkers, which may help to optimize clinical decision algorithms, thereby improving treatment strategies and personalized medicine in patients affected by PC. This novel and intriguing observation warrants analysis of a larger series employing additional markers to dissect the basal and dynamic composition of LEV subpopulations in order to gain a better insight into its potential for liquid biopsy applications in pancreatic cancer.

## Figures and Tables

**Table 1:** Characteristics of the overall patient cohort (*n* = 56).

Overall Study Cohort (*n* = 56)	
Variable	Frequency (%)
Primary Tumor Location	
Head	36 (33.0)
Body/Tail	17 (15.6)
Isthmus	3 (2.8)
Clinical Stage	
Stage I–III	25 (44.6)
Stage IV	31 (55.4)
Tumor grading	
1	2 (3.6)
2	21 (37.5)
3	9 (16.1)
Missing	24 (42.9)
Primary Treatment	
Surgery	9 (16.1)
Systemic Therapy (Borderline Resectable)	15 (26.7)
Systemic Therapy (Locally Advanced/Metastatic)	32 (57.1)
Systemic Therapy	
Gemcitabine + Nab-paclitaxel	29 (51.8)
FOLFIRINOX	12 (21.4)
Gemcitabine	2 (3.6)
FOLFIRI	1 (1.8)
PAX-G	1 (1.8)
Not evaluable/Missing	11 (19.6)

**Table 2:** Characteristics of patients with locally advanced or metastatic PC (*n* = 32).

Locally Advanced/Metastatic Cohort (*n* = 32)	
Variable	Frequency (%)
Line of therapy	
1	29 (90.6)
2	2 (6.3)
≥3	1 (3.1)
Number of metastatic sites	
0	1 (3.1)
1	23 (71.9)
2	5 (15.6)
≥3	3 (9.4)
Liver Metastasis	
Yes	20 (64.5)
No	11 (35.4)
Peritoneal Metastasis	
Yes	8 (25.0)
No	23 (71.9)
Lung Metastasis	
Yes	5 (16.1)
No	26 (83.9)

**Table 3:** Comparison of total and subtype EV concentrations between PC patients (*n* = 56) and age and sex-matched healthy controls (HCs) (*n* = 48).

	PC (*n* = 56)	HC (*n* = 48)	*p*-Value
Age (%)			
≥65	34 (60.7)	22 (39.3)	0.17
<65	22 (39.3)	26 (54.2)	
Sex (%)			
Male	28 (50.0)	31 (52.5)	0.17
Female	28 (50.0)	17 (37.8)	
Total EVs/µL (95% CI)	2168.6 (1940.9–2576.5)	1559.4 (1339.5–1985.5)	0.0003
Leukocyte-derived (CD45+) EVs/µL (95% CI)	280.0 (229.5–343.9)	182.1 (167.4–233.3)	0.001
PD-L1+ EVs/µL (95% CI)	87.5 (70.0–115.6)	58.7 (42.8–82.9)	0.01
PD-L1+CD45+ EVs/µL (95% CI)	68 (47.0–96.1)	44.1 (26.0–57.6)	0.008
PD-L1+CD45- EVs/µL (95% CI)	22.6 (13.5–30.2)	18.1 (11.2–23.0)	0.06

**Table 4:** Univariate and multivariate Cox proportional hazards model predicting OS in the cohort of patients with borderline resectable, locally advanced, or metastatic PC (*n* = 47).

	Univariate Analysis		Bootstrap Results (1000 Replicas)				Multivariate Analysis ^1^	
Variable	HR (95% CI)	*p*.	Bias	SE	95 % CI	*p*.	HR (95% CI)	*p*.
Total EVs								
<2710 EVs/µL	1 [reference]							
>2710 EVs/µL	0.20 (0.05–0.90)	0.04	0.15	0.98	−4.01 to −0.23	0.04		
CD45+ EVs								
<379.1 EVs/µL	1 [reference]							
>379.1 EVs/µL	0.14 (0.03–0.65)	0.01	−0.42	1.02	−4.47 to −0.77	0.01	0.17 (0.04–0.79)	0.02
PD-L1+ EVs								
<124.8 EVs/µL	1 [reference]							
>124.8 EVs/µL	0.21 (0.06–0.75)	0.02	−0.18	0.84	−3.99 to −0.39	0.02		
PD-L1+CD45+ EVs								
<108.5 EVs/µL	1 [reference]							
>108.5 EVs/µL	0.18 (0.04–0.78)	0.02	−0.16	0.96	−4.09 to −0.39	0.02		
PD-L1+CD45- EVs								
<42.7 EVs/µL	1 [reference]							
>42.7 EVs/µL	0.26 (0.07–0.94)	0.04	−0.24	0.88	−3.98 to −0.27	0.01		
ECOG PS								
0	1 [reference]							
1–2	1.78 (0.70–4.55)	0.22	0.07	0.53	−0.33 to −1.80	0.20		
Age (years)								
≥65	1 [reference]							
<65	0.85 (0.31–2.29)	0.75	−0.01	0.57	−1.36 to 0.86	0.74		
No. of metastatic sites								
>1	1 [reference]							
1	0.71 (0.19–2.59)	0.60	0.04	0.97	−1.76 to 3.12	0.59		
BMI								
Continuous Variable	0.89 (0.79–1.00)	0.07	−0.01	0.07	−0.29 to −0.01	0.06		
CA 19.9								
Continuous Variable	1.00 (1.00–1.00)	0.14	−0.00	0.00	−0.001 to 0.00	0.12		
Tumor Grading								
1–2	1 [reference]							
3	0.85 (0.31–2.29)	0.75	−0.01	0.57	−1.37 to 0.87	0.75		
Primary tumor location								
Body/Isthmus/Tail	1 [reference]							
Head	2.03 (0.66–6.22)	0.21	0.03	0.80	−0.54 to 2.59	0.28		
Clinical Stage								
Stage I–II	1 [reference]							
Stage III–IV	1.26 (0.29–5.59)	0.75	0.28	1.40	−1.51 to 3.41	0.80		
Liver Metastasis								
Yes	1 [reference]							
No	1.95 (0.74–5.16)	0.18	0.01	0.48	−0.29 to 1.66	0.12		
Peritoneal Metastasis								
Yes	1 [reference]							
No	0.24 (0.07–0.77)	0.02	−0.48	2.00	−9.58 to 0.00	0.008 ^2^		
NLR								
NLR > 5	1 [reference]							
NLR < 5	0.24 (0.08–0.74)	0.01	−0.29	1.77	−7.73 to 0.00	0.008	0.16 (0.08–0.76)	0.02

^1^ Variables with *p* < 0.05 in the univariate analysis were included in the multivariate analysis. ^2^ Based on 997 samples; Abbreviations: HR: Hazard ratio; SE: standard error; CI: confidence interval.

## Data Availability

The data that support the findings of this study are available from the corresponding author, D.B. (Davide Brocco), upon reasonable request.

## Supplementary Materials

The following supporting information can be downloaded at: https://www.mdpi.com/article/10.3390/cancers14194748/s1, Supplementary Table S1. List of flow cytometry specificities and reagents; Supplementary Figure S1. Gating strategy for EV identification and subtyping in peripheral blood samples. All events were represented on a forward scatter-H/side scatter-H dot-plot and a “platelet-free area” (Morph) region was identified by using platelets as a reference population and gating events with physical parameters lower than platelets (a). The “platelet-free area” (Morph) was represented on a lipophilic cationic dye (LCD)-H/phalloidin-H dot-plot; total EVs were detected as LCD-positive/phalloidin negative events (b). Total EVs were analyzed on a CD45-H/CD133-H dot-plot and CD45+ events (CD45+ EVs) were gated (c). Total EVs were plotted on a CD45-H/PD-L1-H dot-plot and CD45+PD-L1+ and CD45-PD-L1+ events were identified (e); Supplementary Table S2. Spearman rank correlation coefficients between blood-circulating EVs and selected clinical–pathological factors in patients with PC (*n* = 56); Supplementary Figure S2. Box plots showing differences in CD45+PD-L1+ EVs and CD45-PD-L1+ EVs concentrations between patients without liver metastasis and with liver metastasis (a), and between patients with peritoneal metastasis and without peritoneal metastasis (b). Statistical comparison was performed by applying the Mann–Whitney *U* test; Supplementary Figure S3. Overview plots of HRs (with 95% CIs) for all possible cut-off values for blood concentrations of total EVs (a), CD45+ EVs (b), PD-L1+ EVs (c), CD45+PD-L1+ EVs (d), CD45-PD-L1+ EVs (e), as calculated by the Cutoff Finder software. The vertical lines in each plot indicate the optimal cut-offs, which are defined as the point that resulted in the most significant split (log-rank test). Supplementary Table S3. Univariate and multivariate Cox proportional hazards model predicting PFS in the cohort of patients with locally advanced or metastatic PC (*n* = 32); Supplementary Table S4. Univariate exact logistic regression analysis exploring the association between disease control rate (DCR) and baseline blood EV concentrations; Supplementary Table S5. Univariate exact logistic regression analysis exploring the association between disease control rate (DCR) and changes in blood EV concentrations in patients with primary unresectable PC (*n* = 13); Supplementary Figure S4. Histograms showing the distribution of patients achieving (red bars) or not achieving (blue bar) disease control according to variation in LEVs (CD45+ EVs) concentrations. Fisher’s exact test was used to compare changes in EV concentrations and disease control; Table S6: Univariate exact logistic regression analysis exploring the association between disease control rate (DCR) and changes in blood EV concentrations in patients with primary unresectable PC (*n* = 13).
