# Supplementary material for: High Blood Concentration of Leukocyte-Derived Extracellular Vesicles Is Predictive of Favorable Clinical Outcomes in Patients with Pancreatic Cancer: Results from a Multicenter Prospective Study
Source: Cancers (Basel). 2022 Sep 29;14(19):4748. doi: 10.3390/cancers14194748 (PMC9562679; doi:10.3390/cancers14194748)
Supplement: Supplementary file 1 [file cancers-14-04748-s001.zip › cancers-1929838-supplementary.pdf]

Supplementary Table S1. Comparison of age and sex distribution between PC patients (n=56) and healthy controls (n=48).

	PC (n=56)	HC (n=48)	p-value
**Age (%)**			
≥65	34 (60.7)	22 (39.3)	0.17
<65	22 (45.8)	26 (54.2)	
**Sex (%)**			
Male	28 (47.5)	31 (52.5)	0.17
Female	28 (62.2)	17 (37.8)	

Supplementary Table S2. List of flow cytometry specificities and reagents.

Reagent	Fluorochrome/Reagent	Vendor	Clone	Cat. Number	Volume per test (µl)
Lipophilic Cationic Dye (LCD)	-	BD Biosciences	-	626267	0.5
Phalloidin-FITC	FITC	BD Biosciences	-	626267	0.5
CD45	BV510	BD Biosciences	HI30	626266	5
PD-L1	BV421	BD Biosciences	29E.2A3	568320	1
CD133/2	PE	Miltenyi Biotec	293C3	130-113-186	1
EpCAM	PerCP-Cy5.5	BD Biosciences	EBA-1	347199	5

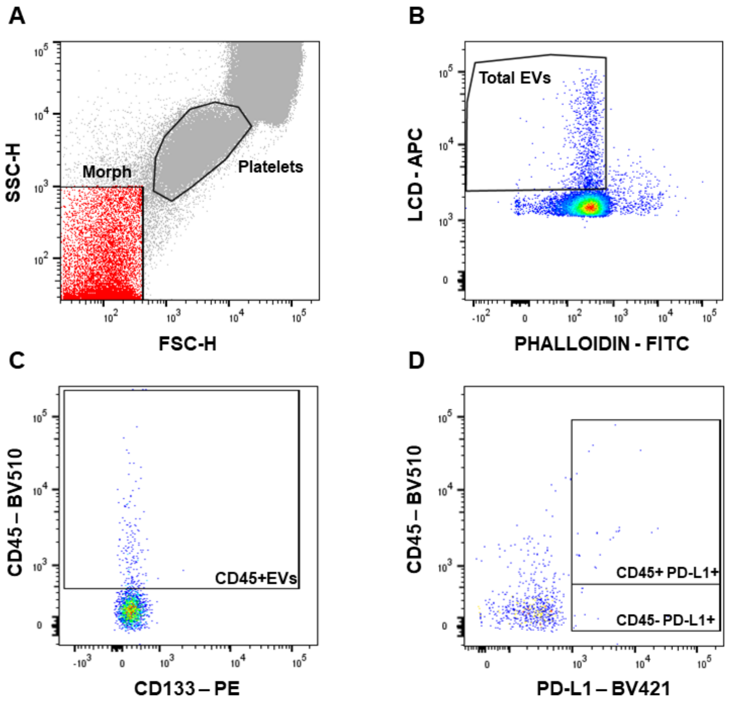

Supplementary Figure S1. Gating strategy for EV identification and subtyping in peripheral blood samples. All events were represented on a forward scatter-H/side scatter-H dot-plot and a "platelet-free area" (Morph) region was identified by using platelets as a reference population and gating events with physical parameters lower than platelets **(A)**. The "platelet-free area" (Morph) was represented on a lipophilic cationic dye (LCD)-H/phalloidin-H dot-plot; total EVs were detected as LCD-positive/phalloidin negative events **(B)**. Total EVs were analyzed on a CD45-H/CD133-H dot-plot and CD45+ events (CD45+ EVs) were gated **(C)**. Total EVs was plotted on a CD45-H/PD-L1-H dot-plot and CD45+PD-L1+ and CD45-PD-L1+ events were identified **(D)**.

Supplementary Table S3. Spearman rank correlation coefficients between blood circulating EVs and selected clinical-pathological factors in patients with PC (n=56).

		**ECOG PS**	**Sex**	**Age**	**BMI**	**Primary Tumor Location**	**Tumor Grading**	**CA 19.9**	**NLR**	**Clinical Stage**	**Number of metastatic sites**	**Liver metstasis**	**Peritoneal metastasis**	**Lung metastasis**
**Total EVs**	Correlation Coefficient	0.009	0.056	0.166	0.132	0.059	-0.021	-0.058	-0.120	-0.048	0.067	0.188	-0.157	-0.108
	p - value (2-tailed)	0.95	0.57	0.09	0.38	0.67	0.91	0.70	0.12	0.73	0.72	0.31	0.40	0.56
**CD45+ EVs**	Correlation Coefficient	0.070	0.120	-0.080	0.000	0.150	-0.030	0.070	-0.190	0.040	0.180	0.320	-0.300	0.070
	p - value (2-tailed)	0.62	0.39	0.61	0.99	0.28	0.89	0.62	0.19	0.78	0.33	0.08	0.10	0.62
**PD-L1+EVs**	Correlation Coefficient	0.070	0.060	0.000	-0.250	0.040	0.170	0.130	0.000	0.020	0.030	**0.377**	**-0.460**	0.070
	p - value (2-tailed)	0.60	0.64	1.00	0.09	0.79	0.38	0.37	0.99	0.86	0.86	**0.04**	**0.01**	0.60
**CD45+ PD-L1+EVs**	Correlation Coefficient	-0.040	0.090	0.070	-0.210	-0.010	0.200	0.090	-0.010	0.040	0.000	**0.437**	**-0.478**	0.070
	p - value (2-tailed)	0.80	0.52	0.63	0.16	0.94	0.28	0.56	0.95	0.75	0.98	**0.01**	**0.01**	0.71
**CD45- PD-L1+EVs**	Correlation Coefficient	-0.100	0.040	0.010	-0.220	0.180	0.140	0.120	-0.110	-0.020	-0.020	0.260	-0.340	0.000
	p - value (2-tailed)	0.51	0.75	0.94	0.15	0.18	0.46	0.43	0.44	0.89	0.91	0.16	0.06	1.00

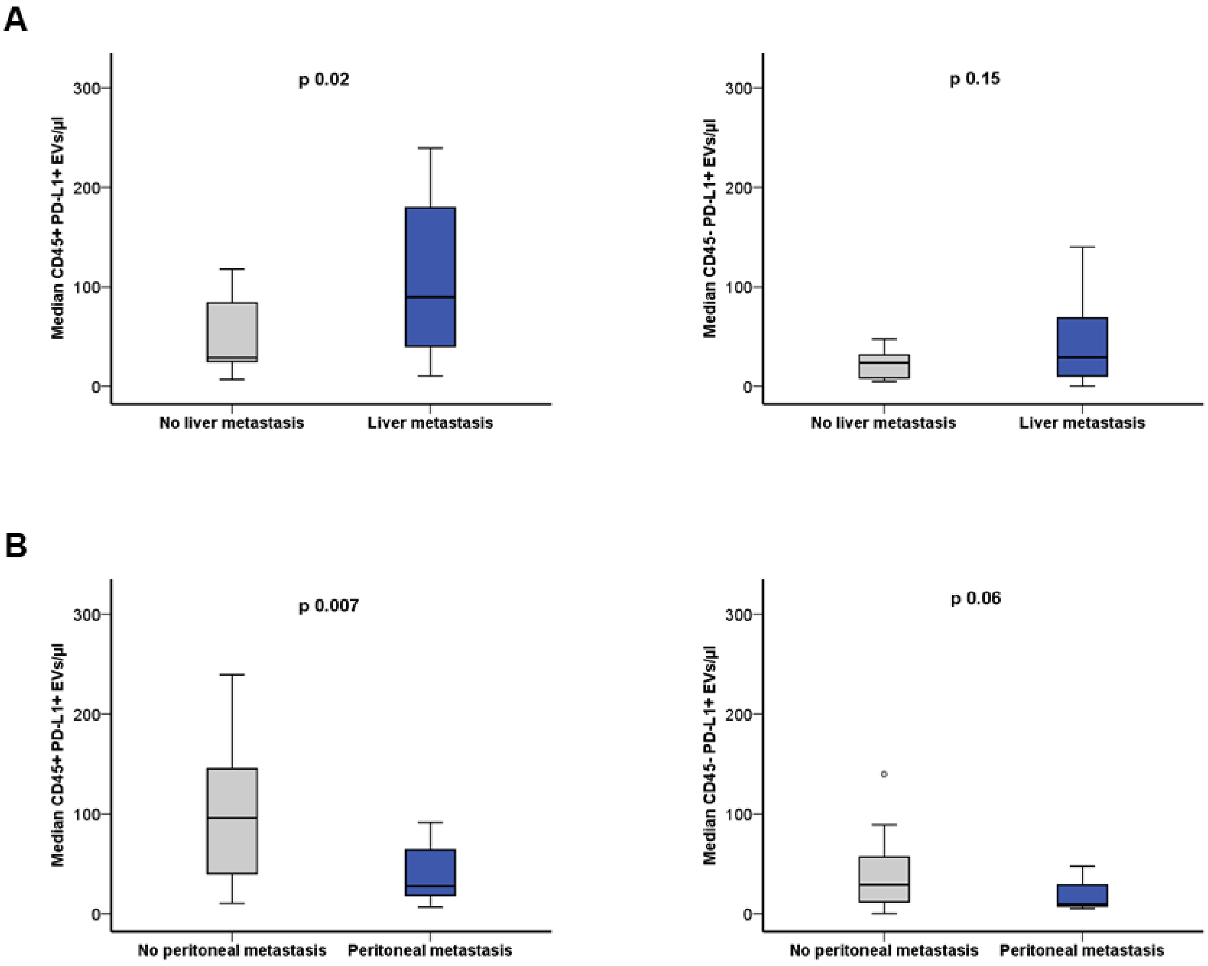

Supplementary Figure S2. Box plots showing differences in CD45+PD-L1+ EVs and CD45-PD-L1+ EVs concentration between patients without liver metastasis and with liver metastasis **(a),** and between patients with peritoneal metastasis and without peritoneal metastasis **(b)**. Statistical comparison was performed by applying the Mann–Whitney U test.

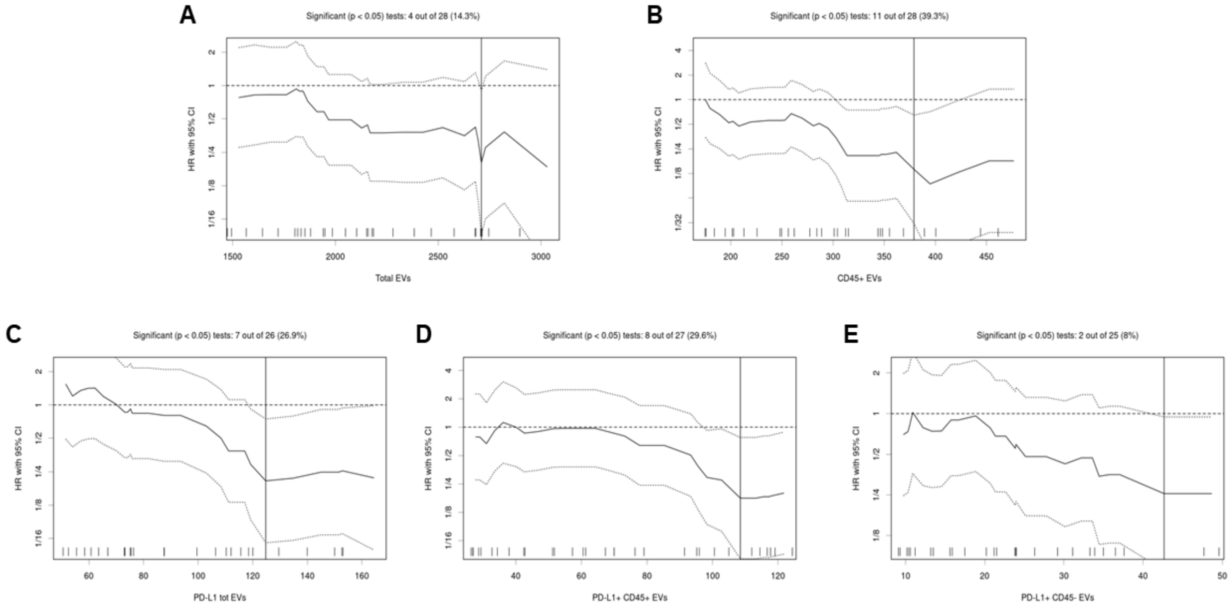

Supplementary Figure S3. Overview plots of HRs (with 95% CIs) for all possible cut-off values for blood concentrations of total EVs **(A)**, CD45+ EVs **(B)**, PD-L1+ EVs **(C)**, CD45+PD-L1+ EVs **(D)**, CD45-PD-L1+ EVs **(E)**, as calculated by the Cutoff Finder software. The vertical lines in each plot indicate the optimal cut-offs, which are defined as the point that resulted in the most significant split (log-rank test).

Supplementary Table S4. Univariate and multivariate Cox proportional hazards model predicting PFS in the cohort of patients with locally advanced or metastatic PC (n=32).

	**Univariate Analysis**		**Bootstrap results (1000 replicas)**				**Multivariate Analysis[1]**	
**Variable**	**HR (95% CI)**	**p.**	**Bias**	**SE**	**95 % CI**	**p.**	**HR (95% CI)**	**p.**
**Total EVs**								
<2710 EVs/µl	1 [reference]							
>2710 EVs/µl	**0.19 (0.04-0.84)**	**0.03**	-0.25	0.99	-4.12 to -0.38	**0.001**		
**CD45+ EVs**								
<379.1 EVs/µl	1 [reference]							
>379.1 EVs/µl	**0.07 (0.01-0.54)**	**0.01**	-0.35[1]	0.97[1]	-4.60 to -1.33[1]	**0.006[1]**	0.10 (0.01-0.82)	**0.03**
**PD-L1+ EVs**								
<124.8 EVs/µl	1 [reference]							
>124.8 EVs/µl	**0.22 (0.06-15.90)**	**0.02**	-0.16	0.86	-4.08 to -0.37	**0.01**		
**PD-L1+CD45+ EVs**								
<108.5 EVs/µl	1 [reference]							
>108.5 EVs/µl	0.31 (0.09-1.07)	0.06	-0.09	0.84	-3.74 to -0.06	0.04		
**PD-L1+CD45- EVs**								
<42.7 EVs/µl	1 [reference]							
>42.7 EVs/µl	**0.21 (0.06-0.77)**	**0.02**	-0.24[1]	0.99[1]	-4.50 to -0.41[1]	**0.009[1]**		
**ECOG PS**								
0	1 [reference]							
1-2	1.12 (0.44-2.86)	0.80	-0.14	0.67	-1.22 to 1.20	0.80		
**No. of metastatic sites**								
>1	1 [reference]							
1	0.77 (0.25-2.37)	0.64	0.002	0.58	-1.56 to 0.98	0.69		
**CA 19.9**								
Continuous Variable	1.00 (1.00-1.00)	0.21	0.00	0.00	0.00 to 0.00	0.26		
**Tumor Grading**								
1-2	1 [reference]							
3	1.54 (0.40-5.89)	0.53	0.10	1.25	-1.18 to -4.36	0.47		
**Chemotherapy regimen**								
FOLFIRINOX	1 [reference]							
Gemcitabine + Abraxane	1.26 (0.38-4.16)	0.70	0.39	2.60	-2.58 to -11.56	0.68		
Gemcitabine alone	5.56 (0.84-36.60)	0.07	1.23	4.43	-1.12 to 14.03	0.07		
**Line of therapy**								
>1st line	1 [reference]							
1st line	0.54 (0.14-1.98)	0.35	-0.04	0.44	-1.59 t0 0.12	0.11		
**Liver Metastasis**								
Yes	1 [reference]							
No	**3.86 (1.14-13.07)**	**0.03**	0.08	0.80	0.32 to 3.06	**0.004**		
**Peritoneal Metastasis**								
Yes	1 [reference]							
No	**0.21 (0.06-0.80)**	**0.02**	-0.24	1.44	-5.46 to -0.35	**0.003**		
**NLR**								
NLR>5	1 [reference]							
NLR<5	**0.14 (0.03-0.56)**	**0.006**	-1.06	2.89	-12.00 to -0.26	**0.003**	0.20 (0.05-0.83)	**0.03**

[1] based on 999 samples; Abbreviations: HR: Hazard ratio; SE: standard error; CI: confidence interval.

Supplementary Table S5. Univariate exact logistic regression analysis exploring the association between disease control rate (DCR) and baseline blood EV concentrations.

Variable		OR	95% CI	p-value
**Total EVs**	High	[reference]		
	Low	0.14	0.00-1.29	0.08
**CD45+ EVs**	**High**	**[reference]**		
	**Low**	**0.11**	**0.00-0.95**	**0.04**
**PD-L1+ EVs**	High	[reference]		
	Low	0.15	0.00-1.89	0.21
**PD-L1+CD45+ EVs**	High	[reference]		
	Low	0.20	0.00-2.58	0.35
**PD-L1+CD45- EVs**	High	[reference]		
	Low	0.21	0.00-2.59	0.35

Abbreviations: OR: odd ratio; CI: confidence interval

Supplementary Table S6. Univariate exact logistic regression analysis exploring the association between disease control rate (DCR) and changes in blood EV concentrations in patients with primary unresectable PC (n=13).

Variable	OR	95% CI	*p*-value
**Fold change of Total EVs**	1.70	0.44-7.36	0.45
**Fold change of CD45+ EVs**	**6152.5**	**1.92-NE**	**0.003**
**Fold change of PD-L1+ EVs**	2.66	0.90-14.6	0.09
**Fold change of PD-L1+CD45+ EVs**	1.69	0.85-5.48	0.17
**Fold change of PD-L1+CD45- EVs**	1.09	0.56-2.16	0.69

Abbreviations: OR: odd ratio; CI: confidence interval.

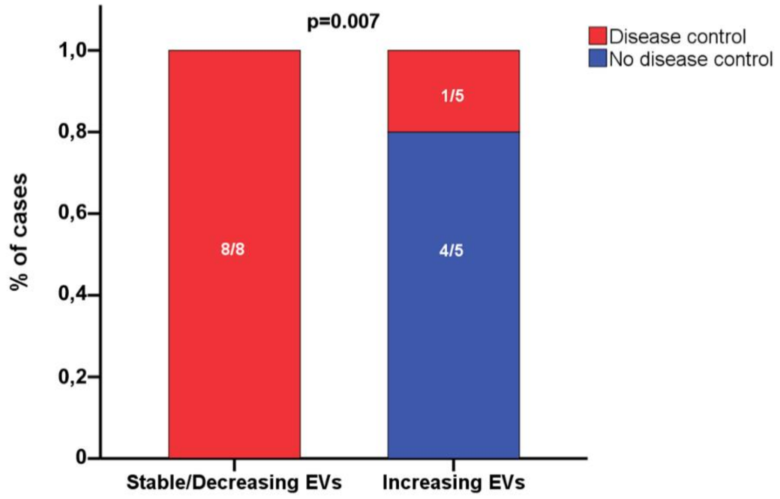

Supplementary Figure S4. Histograms showing the distribution of patients achieving (red bars) or not achieving (blue bar) disease control according to variation of LEVs (CD45+ EVs) concentrations. Fisher's exact test was used to compare change in EV concentrations and disease control.
